# Assessing the validity and reliability of family factors on physical activity: A case study in Turkey

**DOI:** 10.1371/journal.pone.0197920

**Published:** 2018-06-14

**Authors:** Sharalyn Steenson, Hilal Özcebe, Umut Arslan, Hande Konşuk Ünlü, Özgür M. Araz, Mahmut Yardim, Sarp Üner, Nazmi Bilir, Terry T.-K. Huang

**Affiliations:** 1 University of Nebraska Medical Center College of Public Health, Omaha, NE, United States of America; 2 Hacettepe University Institute of Public Health, Ankara, Turkey; 3 University of Nebraksa–Lincoln College of Business Administration, Lincoln, NE, United States of America; 4 City University of New York Graduate School of Public Health and Health Policy, New York, NY, United States of America; IRCCS E. Medea, ITALY

## Abstract

**Background:**

Childhood obesity rates have been rising rapidly in developing countries. A better understanding of the risk factors and social context is necessary to inform public health interventions and policies. This paper describes the validation of several measurement scales for use in Turkey, which relate to child and parent perceptions of physical activity (PA) and enablers and barriers of physical activity in the home environment.

**Method:**

The aim of this study was to assess the validity and reliability of several measurement scales in Turkey using a population sample across three socio-economic strata in the Turkish capital, Ankara. Surveys were conducted in Grade 4 children (mean age = 9.7 years for boys; 9.9 years for girls), and their parents, across 6 randomly selected schools, stratified by SES (n = 641 students, 483 parents). Construct validity of the scales was evaluated through exploratory and confirmatory factor analysis. Internal consistency of scales and test-retest reliability were assessed by Cronbach’s alpha and intra-class correlation.

**Results:**

The scales as a whole were found to have acceptable-to-good model fit statistics (PA Barriers: RMSEA = 0.076, SRMR = 0.0577, AGFI = 0.901; PA Outcome Expectancies: RMSEA = 0.054, SRMR = 0.0545, AGFI = 0.916, and PA Home Environment: RMSEA = 0.038, SRMR = 0.0233, AGFI = 0.976). The PA Barriers subscales showed good internal consistency and poor to fair test-retest reliability (personal α = 0.79, ICC = 0.29, environmental α = 0.73, ICC = 0.59). The PA Outcome Expectancies subscales showed good internal consistency and test-retest reliability (negative α = 0.77, ICC = 0.56; positive α = 0.74, ICC = 0.49). Only the PA Home Environment subscale on support for PA was validated in the final confirmatory model; it showed moderate internal consistency and test-retest reliability (α = 0.61, ICC = 0.48).

**Discussion:**

This study is the first to validate measures of perceptions of physical activity and the physical activity home environment in Turkey. Our results support the originally hypothesized two-factor structures for Physical Activity Barriers and Physical Activity Outcome Expectancies. However, we found the one-factor rather than two-factor structure for Physical Activity Home Environment had the best model fit. This study provides general support for the use of these scales in Turkey in terms of validity, but test-retest reliability warrants further research.

## Introduction

Obesity rates in both children and adults have been rising around the world. The rising levels of obesity in developing countries—some now outpacing those in developed countries—is of particular concern.[1] Middle Eastern and Eastern European countries have been shown to have some of the highest prevalence rates of childhood overweight and obesity among developing nations.[1] In Saudi Arabia, overweight prevalence in male children (ages 6–18 years) was at 11.2%, and obesity at 15.8%.[1] In Lebanon boys ages 6–8 years, the prevalence of overweight was 26% and obesity was 7%, while the rates in girls were 25% and 6%, respectively.[1] In Turkey, recent estimates placed the prevalence of overweight and obesity in youth 10–19 years at 18.3%,[2] and in certain areas of the country nearly one in four children aged 6–16 years was found to be overweight or obese.[3] Similarly, the Childhood Obesity Surveillance Initiative (COSI) found in a nationally representative sample that the prevalence of overweight and obesity in 7-8-year-old Turkish children was 14.2% and 8.3%, respectively.[4] However, our most recent study among children in Ankara–the second largest city in Turkey–suggests that the prevalence of overweight (21.2%) and obesity (14.6%) may be much higher in large metropolitan regions within Turkey.[5]

Current data shows significant differences in the prevalence of adult overweight and obesity between urban and rural areas in Turkey,[6] with urban children having a higher risk of becoming overweight and obese. One study estimated that in Turkish urban children aged 10–19 years, over one in five was obese, which was twice the rate seen in this age group for rural areas.[3] In addition, in COSI, 9.6% of younger urban children aged 7–8 years were obese compared to 3.3% in rural areas.[4] These findings indicate a significant need for studies to improve our understanding of factors that contribute to the high prevalence of childhood obesity as well as potential intervention strategies in urban communities.

Complex behavioral, social, and environmental changes interact to promote the development of obesity,[7] and international childhood obesity research has highlighted the need to address these multiple levels of factors that contribute to the obesity epidemic.[8] The social context surrounding the development of obesity in middle income countries such as Turkey is not well understood, and research in this area is needed to help guide public health interventions and policy.[7] Understanding the socio-cultural environment in which obesity is perceived is essential to designing effective obesity interventions.[7,9]

Childhood obesity has been shown to increase the risk of chronic diseases in adulthood such as cardiovascular disease, type 2 diabetes, and certain cancers.[10] The health behaviors of the parents and the home food and physical activity environment all influence children’s lifestyle and habits significantly.[11] In a number of studies, parental overweight or obesity has been shown to be an independent risk factor for child overweight and obesity, likely due to a combination of genetic and environmental factors.[12–16]

There are a number of barriers in the home, neighborhood, and school environments that can inhibit physical activity. Perceived barriers such as lack of access, weather, safety, etc. have been shown to reduce the level of physical activity in high school students and adults.[17,18] Research on parents shows that similar issues such as lack of social support, competing priorities for time, and financial concerns act as barriers to their ability to promote healthy behaviors and weight at home for their children.[19] In one study, parents who reported a lack of easy access to outdoor play areas for their daughters also reported lower use of active transportation by their daughters (i.e., walking, biking).[20]

Outcome expectations are personal factors within Bandura’s Social Cognitive Theory,[21] which influence health behaviors in people- the more positive the outcome expectations are, the more likely the person will be to engage in that behavior.[22] In children, their beliefs about the positive or negative results of performing a particular health behavior (outcome expectancies) have been shown to be related to perceived benefits and attitudes,[23] as well as to have the ability to modify self-regulatory skills for maintenance of behavior change.[24] This is consistent with the review conducted by the World Health Organization (WHO) that reported that correlates of youth physical activity include perceived benefits and attitudes.[8]

The home and family environment has also been shown to affect physical activity levels in children. Parenting practices and behaviors related to food and physical activity have been linked to the development and establishment of health behaviors among children, which ultimately contributes to their risk of obesity.[25] Specifically, parental support for physical activity can influence physical activity levels in children. Children who receive more parental support from parents to be physically active (encouragement, transportation, shared activities) reported higher levels of physical activity.[26–30]

Information regarding these factors in Turkey and other middle-income countries is limited, but some evidence exists showing a significant relationship between parental and child obesity.[31] This paper is part of a larger study, the Childhood Obesity Study of Ankara (COSA), a population study across three socio-economic strata in the Turkish capital, Ankara. In this paper, we aim to validate several measurement scales in Turkey that have been previously validated in other countries: the Parent Physical Activity Barriers scale,[17] the Child Physical Activity Outcome Expectancies Scale,[32] and the Child Physical Activity Home Environment scale.[33] These scales relate to child perceptions of physical activity and enablers and barriers of physical activity in the home environment. The validation of an existing psychological instrument in a new population is a vital step in the process of adaptation. Validation of an existing tool allows the researchers to ensure the tool is culturally appropriate, and that the meaning and difficulty of the items are suitable and conceptually equivalent to the original. This ultimately allows for easier comparisons between populations and a greater ability to generalize findings.[34] Validation of these scales in Turkey will further research on family factors in obesity and the design and testing of interventions targeting these risk factors.

## Methods

### Partnership

In order to facilitate research in this area, a unique partnership was formed between the University of Nebraska Medical Center in the US and Hacettepe University Institute of Public Health in Ankara, Turkey. A memorandum of understanding was signed by the two public health institutions in the fall of 2013 as part of a broader collaboration agreement between the two institutions. This collaboration promotes the advancement of obesity and health research in Turkey and globally. This project was approved by the Non-interventional Clinical Researches Ethics Board of Hacettepe University, Ankara, Turkey.

### Research setting

In this study, a population-based random stratified survey of 641 students and 537 parents in three socioeconomic strata (SES) in Ankara was conducted that included individual and family psychosocial and behavioral risk factors related to the development of childhood overweight and obesity and that may be associated with parental support.

### Study design

Investigators from the University of Nebraska Medical Center assisted with survey development, followed by survey administration by the investigators at Hacettepe University to parents and children through local schools. Measures from the existing literature were adapted and translated, and then back-translated. The survey instruments were then piloted in a dozen parent-child dyads in a school not part of the study to gauge feasibility and time requirement. Participants were asked to complete the survey and interviewed to determine any issues with the survey translation or adaptation (e.g., is the survey wording clear, are response options compatible with participant experiences, etc.). Subsequently, the surveys were examined by Turkish linguists to fine-tune the language. After the establishment of survey language and feasibility, the surveys were administered across 6 randomly selected schools to children in grade 4 (9-10-years-old) and their parents, stratified by SES (n = 641 students, 537 parents).

### Data collection & measurement

In this study, a stratified random sampling design was used. Stratification of the primary schools in Ankara was achieved by ranking counties according to SES level (low-middle-high), based on previously reported socio-economic indicators and social structures.[35] The high SES stratum consisted exclusively of the private schools, with the public schools of Cankaya and Yenimahalle counties forming the middle SES, and the lower SES stratum was formed by public schools from Altındağ, Mamak and Sincan counties. The sampling unit within each stratum was 4th grade classrooms. This validation study began with the completion of survey translations and user testing of final survey instruments for parents and Grade 4 children (mean age = 9.7 years for boys; 9.9 years for girls), followed by selection of approximately 650 parent-child dyads from randomly selected schools in order to assess test-retest reliability and validate the scale. Within each school, a minimum of 80–100 students were recruited into the study via the random selection of 2–5 classrooms by taking into account density of classrooms of the school. All classes were included in some schools if the number of Grade 4 students were below 80. The validation surveys were administered to parent-child dyads twice over a 3-week interval to assess test-retest reliability. The surveys were given at six schools, including 2 in each SES category. Each school sent information regarding the study and informed consent to parents, and passive student assent was sought. For both administrations, children were given a packet with the child survey, which was filled out at school, and the parent survey, which was taken home and asked to be returned within 3 days. The surveys were labeled with unique survey numbers, as well as the individual students’ identification numbers. No physical measurements were taken in phase I validation surveys. Parent and child surveys were evaluated separately and were not matched for the analysis performed in the current paper. The results from Phase I helped to inform the full implementation (Phase 2) of COSA in 46 schools, in which parent and child data are being matched and analyzed together (not discussed in this paper).

### Measurement of constructs

#### Physical activity barriers (parent survey)

Barriers such as lack of access, weather, safety, etc. have been shown to inhibit physical activity. Timperio et. al. showed that in parents who reported that their daughters were not able to easily access play areas, the girls were less likely to use active transportation (walking, biking) to get to local recreation areas.[20] The validity and reliability of a 16-item scale measuring perceived barriers to physical activity scale in high school students was initially measured by Allison et al (1999).[18] The scale was found to have a two factor structure- composed of personal/individual barriers, and social/environmental barriers. Salmon et. al also explored the association between physical activity level and perceived barriers, validating a modified 13-item version of the Allison scale in adults (α = 0.73).[17] While Salmon et al discussed the scale as having two factors, analysis was performed only on the scale as a whole. In the present study, the previously validated 13-item Likert scale[17] was used to evaluate the parents’ perceived barriers to physical activity for their children. Responses ranging from *(1) not a barrier* to *(5) very much a barrier* were used to evaluate the following potential personal and environmental physical activity barriers: *cost*, *weather*, *safety*, *pollution*, *no access*, *no sidewalk*, *age*, *disability or injury*, *tired*, *lack of time*, *work commitments*, *family commitments*, and *other priorities*.

#### Physical Activity Outcome Expectancies (child survey)

The construct of physical activity outcome expectancies refers to the motivational determinants shown to influence physical activity level. The validity and reliability of psychosocial measures examining outcome expectancies for physical activity in 8-11-year-old African American girls was shown in the 17-item Outcome Expectancies Likert scale in the *Girls health Enrichment Multisite Study* (GEMS).[32] This scale was further divided into positive and negative outcome expectancies. For the Positive Outcome expectancy measure, the internal consistency estimate was α = 0.72 and the test-retest reliability was r = 0.22. For the Negative Outcome Expectancy measure, the internal consistency estimate was α = 0.68 and the test-retest reliability was r = 0.38. Positive outcome expectancies were measured by participant selection of *(1) true of me; (2) sort of true of me;* or *(3) not true of me* as responses to the following questions: “doing physical activity will…” *make me stronger; keep me from gaining weight; teach me about health and fitness; make me look better; help me to have more energy; make me better at sports; be fun to do with my friends;* and *be fun*. Negative outcome expectancy statements included: *make me feel like I am not as good at sports as other kids; make others tease me; make me too tired; make me feel clumsy; be hard because I am often chosen last to be on a team; take too much time; cause me to get hurt; mess up my hair;* and *make me sweat too much*.

#### Physical activity home environment (child survey)

In addition to outcome expectancies, the effect of the home environment on physical activity among 8–11 year old African American girls was also explored in the *Girls health Enrichment Multisite Study* (GEMS).[33] Previous studies have shown that girls who lived in more physical activity promoting environments, such as those with access to safe play spaces and sports equipment, reported higher physical activity levels.[28] In the present study, the role of the home environment was investigated in students through the students’ perception of parent support through a 2-item scale looking at parent permissiveness for sedentary activities (α = 0.86) and a 5-item scale looking at the students’ perception of parent support for physical activity at home (α = 0.90). For the subscale on parental permissiveness of sedentary activities, the participants rated their response from *(1) almost never* to *(3) almost always* for the following statements: *My parent(s) or other adult allows me to watch as much TV as I want;* and *my parent(s) or other adult allows me to play video and computer games as much as I want*. For the subscale on parental support of physical activity, the participants rated their response from *(1) almost never* to *(3) almost always* for the following statements: *It is safe to play outside where I live; my parent(s) or other adult tries to get me to play outside when it is nice; my parent(s) or other adult tries to get me to be physically active instead of watching TV; my parent(s) or other adult goes for walks with me;* and *my family is physically active*.

### Data analysis

Statistical analysis was performed using IBM SPSS v. 23 and IBM AMOS v. 23. For this validation study, initial analysis included descriptive measures (means, frequencies, etc.) for all measures including demographics, variables, and scales. Dependent variables and scales were assessed for outliers and tested for normality using visual assessments and the Kolmogorov-Smirnov test. As needed, measures were transformed to normal distributions. Scale validity was assessed using exploratory and confirmatory factor analysis. The initial round of surveys (test) were used for the exploratory factor analyses, and then the confirmatory factor analyses were done with the second round of surveys (re-test). Varimax rotation was used in exploratory factor analyses for Physical Activity Barriers and Physical Activity Outcome Expectancies scales, but factors were allowed to correlate in confirmatory factor analyses. Rotated factor loadings of at least 0.32 were considered to be significant (using a two-tailed alpha of 0.01). [36]

Model fit was determined using the following statistical tests: root mean square error of approximation (RMSEA) for closeness of fit (good fit = <0.06, acceptable = <0.08) [37,38], standardized root mean square residual (SRMR) (good fit = < .05, acceptable = <0.08)[37] to determine the difference between the sample covariance matrix and the model covariance matrix, and adjusted goodness of fit index (AGFI) (good fit = >0.9)[37] to determine the proportion of variance accounted for by the model. Internal consistency of scales and test-retest reliability were assessed by Cronbach’s alpha, intra-class correlation (ICC), and Spearman’s correlation coefficient.

## Results

The dataset consisted of 641 students (n = 345 boys and 296 girls) and 483 parents (n = 108 male and 375 female) that completed both surveys in the three-week period. The age range for the students was 9–10 years (mean age = 9.7 years for boys; 9.9 years for girls), with 38% coming from low, 32% from middle, and 30% from high SES households. The mean age of parents was 37.6 ± 5.7 years (male: 36.6 ± 5.4, female: 40.8 ± 5.8), with 38% coming from low, 36% from middle, and 25% from high SES households. Descriptive statistics are shown in Table 1.

**Table 1:** Demographics of study population.

Child	N (641)	%	Age (years) Mean± SD	Parent	N (483)	%	Age (years) Mean± SD
**Gender**				**Gender**			
Male	345	53.8	9.7 ± 2.1	Male	108	22.0	36.6 ± 5.4
Female	296	46.2	9.9 ± 1.4	Female	375	78.0	40.8 ± 5.8
**SES**				**SES**			
Low	243	38.0		Low	184	38.1	
Middle	205	32.0		Middle	178	36.9	
High	193	30.0		High	121	25.1	

### Physical activity barriers (Parent)

**Fig 1:**
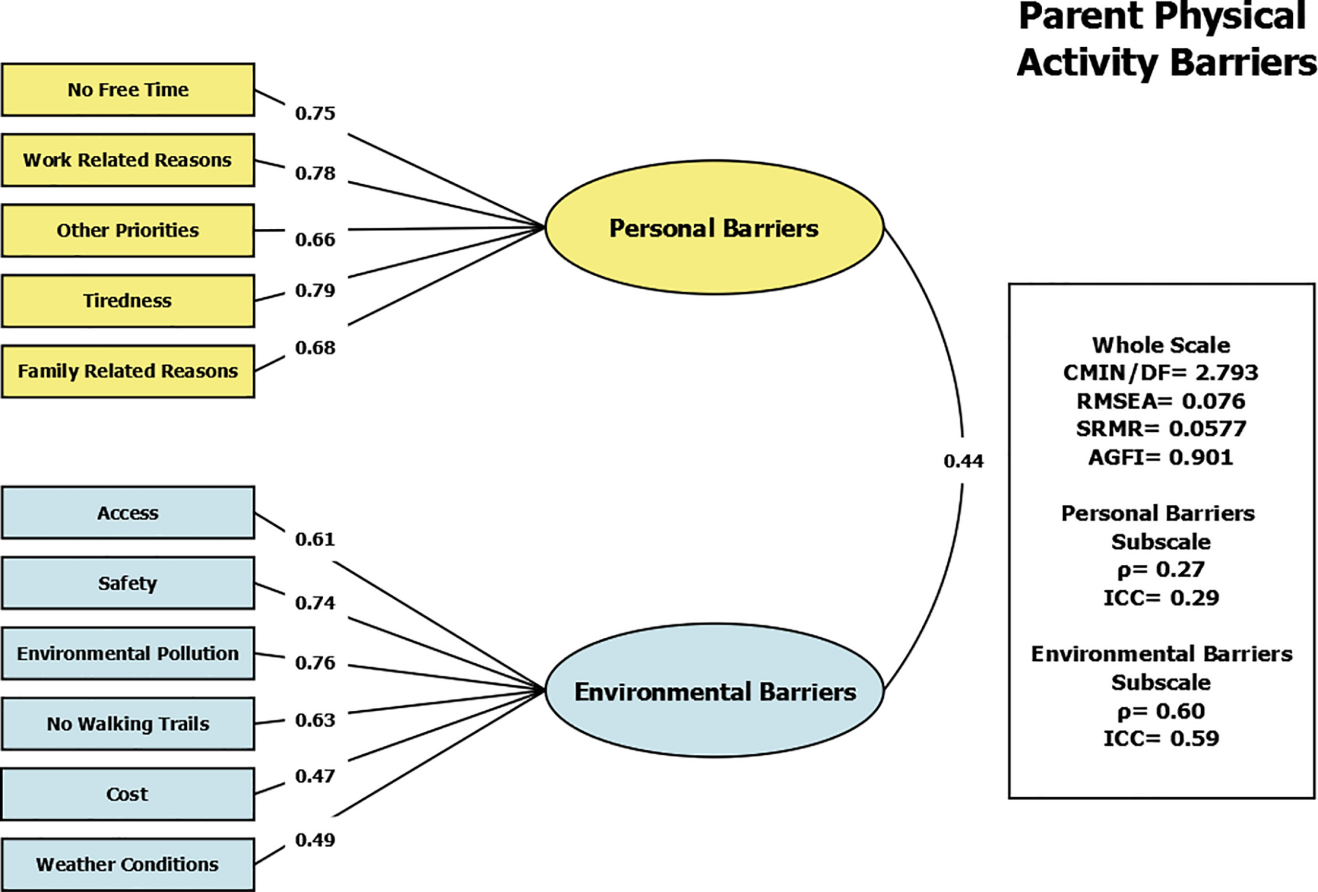
Confirmatory factor analysis of the Physical Activity Barriers Scale (Parent) showing model fit statistics and individual factor loadings for two dimensions—personal barriers and environmental barriers.

The construct of physical activity barriers was measured in the parents. The Physical Activity Barriers scale contained 11 items. Exploratory factor analysis yielded two factors: personal barriers (Internal consistency: α = 0.79, test re-test reliability: ICC = 0.29, ρ = 0.27), and environmental barriers (Internal consistency: α = 0.73, test re-test reliability: ICC = 0.59, ρ = 0.60). Factor loadings are shown in Table 2. The confirmatory model (Fig 1) had a RMSEA value of 0.076, SRMR of 0.0577, and an AGFI of 0.901.

**Table 2:** Individual item factor loadings in Turkish and English for the Physical Activity Barriers Scale (Parent) showing two dimensions.

Turkish	English	Component	
		1	2
Zamanın olmaması	No free time	.815	
İşe bağlı nedenler	Work-related reasons	.770	
Diğer öncelikler	Other priorities	.719	
Yorgunluk	Tiredness	.684	
Aileye bağlı nedenler	Family-related reasons	.670	
Olanaklara erişememe	No access to facilities/ resources		.742
Güvenlik	Safety		.732
Çevre kirliliği	Environmental Pollution		.728
Yürüyüş yollarının olmaması	No walking trails		.581
Maliyet	Cost		.572
Hava durumu	Weather conditions		.516

Rotation Method: Varimax rotation

### Physical Activity Outcome Expectancies (Child)

**Fig 2:**
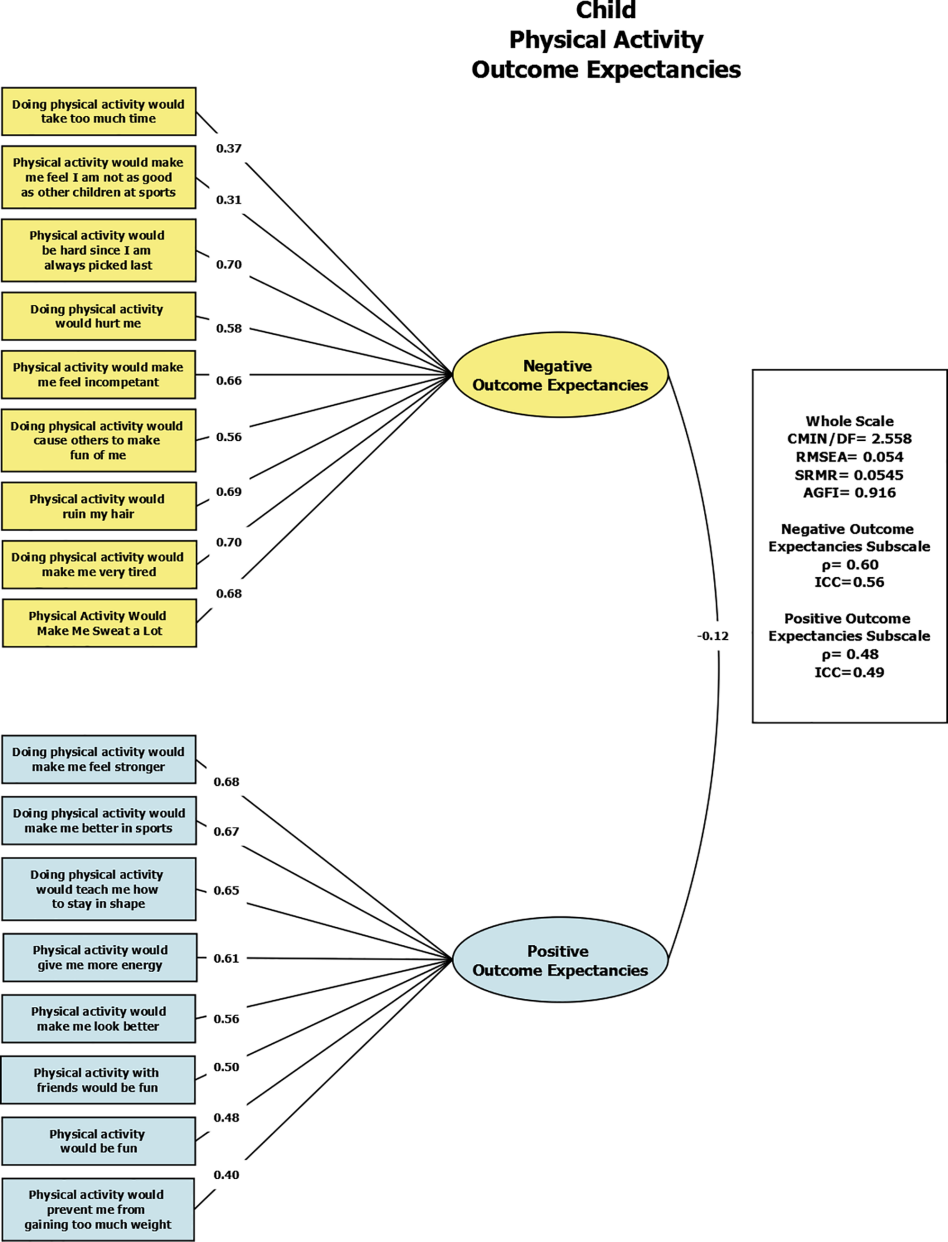
Confirmatory factor analysis of the Physical Activity Outcome Expectancies Scale (Child) showing model fit statistics and individual factor loadings for two dimensions—negative outcome expectancies and positive outcome expectancies.

Using exploratory factor analysis, the scale of Physical Activity Outcome Expectancies was measured in the students. The scale contained 17 items. Exploratory factor analysis found two factors in this scale: negative outcome expectancies (α = 0.77, ICC = 0.56, ρ = 0.60) and positive outcome expectancies (α = 0.74, ICC = 0.49, ρ = 0.48). Table 3 shows the factor loadings. The confirmatory model (Fig 2) showed a RMSEA value of 0.054, SRMR of 0.0545, and an AGFI of 0.916. In addition, when separated by gender the data showed similar results, again having two factors: negative outcome expectancies (boys: α = 0.78, ICC = 0.53, ρ = 0.59; girls: α = 0.72, ICC = 0.58, ρ = 0.58) and positive outcome expectancies (boys: α = 0.74, ICC = 0.45, ρ = 0.48; girls: α = 0.75, ICC = 0.55, ρ = 0.50). For boys the confirmatory model showed a RMSEA value of 0.064, SRMR of 0.0669, and an AGFI of 0.875, and for girls it showed a RMSEA value of 0.056, SRMR of 0.0606, and an AGFI of 0.887.

**Table 3:** Individual item factor loadings in Turkish and English for the Physical Activity Outcome Expectancies Scale (Child) showing two dimensions.

Turkish	English	Component	
		1	2
Fiziksel etkinlik yapmak çok zaman alacaktır	Doing physical activity would take too much time.	.669	
Sıklıkla takıma en son seçilen kişi ben olduğum için fiziksel etkinlik yapmak zor olacaktır	It would be hard to do physical activity, since I am often the last one to be selected in the team.	.587	
Fiziksel etkinlik yapmak bana fiziksel zarar verecektir	Doing physical activity would hurt my body.	.685	
Fiziksel etkinlik yapmak kendimi yetersiz hissetmeme neden olacaktır	Doing physical activity would make me feel incompetent.	.658	
Fiziksel etkinlik yapmak, diğerlerinin benimle dalga geçmesine neden olacaktır	Doing physical activity would make others make fun of me.	.647	
Fiziksel etkinlik yapmak saçımı bozacaktır	Doing physical activity would ruin my hair.	.588	
Fiziksel etkinlik yapmak beni çok yorgun hissettirecektir	Doing physical activity would make me feel very tired.	.605	
Fiziksel etkinlik yapmak beni çok terletecektir	Doing physical activity would make me sweat a lot.	.485	
Fiziksel etkinlik yapmak, benim sporda diğer çocuklar kadar iyi olmadığımı hissettirecektir	Doing physical activity would make me feel like I am not as good as the other children in sports.	.423	
Fiziksel etkinlik yapmak kendimi daha güçlü hissettirecektir	Doing physical activity would make me feel stronger.		.732
Fiziksel etkinlik yapmak sporda daha iyi olmamı sağlayacaktır	Doing physical activity would make be better in sports.		.667
Fiziksel etkinlik yapmak sağlıklı ve formda olma ile ilgili pek çok şey öğretecektir	Doing physical activity would teach me many things about health and being in good shape.		.689
Fiziksel etkinlik yapmak daha çok enerjim olmasını sağlayacaktır	Doing physical activity would make me have more energy.		.538
Fiziksel etkinlik yapmak daha iyi görünmemi sağlayacaktır	Doing physical activity would make me look better.		.608
Arkadaşlarla beraber fiziksel etkinlik yapmak eğlenceli olacaktır	Doing physical activity with friends would be fun.		.448
Fiziksel etkinlik yapmak eğlenceli olacaktır	Doing physical activity would be fun.		.481
Fiziksel etkinlik yapmak çok fazla kilo almamı engelleyecektir	Doing physical activity would prevent me from gaining too much weight.		.620

Rotation Method: Varimax rotation

### Physical Activity Home Environment (Child)

**Fig 3:**
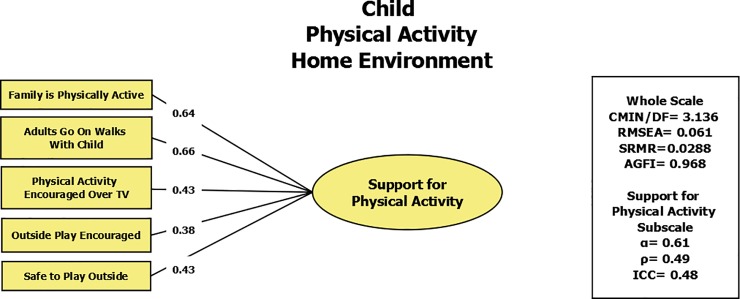
Confirmatory factor analysis of the Physical Activity Home Environment Scale (Child) showing model fit statistics and individual factor loadings for two dimensions—support for physical activity and permissiveness for sedentary activities.

Using exploratory factor analysis, the construct of Physical Activity Home Environment was evaluated in the students. The scale contained 7 items and was shown to have two factors: support for physical activity (α = 0.65, ICC = 0.48 ρ = 0.49), and permissiveness for sedentary activities (α = 0.55, ICC = 0.55, ρ = 0.40), with good model fit statistics (RMSEA = 0.038, SRMR = 0.0288, AGFI = 0.976). However, the confirmatory model (Fig 3) showed that the model fit best with only one factor in terms of support for physical activity, with 5 items (RMSEA value of 0.061, SRMR of 0.0288, and an AGFI of 0.968). The factor loadings are shown in Table 4. When both factors were included, this resulted in a standardized estimate larger than 1 (1.58) and negative variance in the model for one of the items loading on permissiveness for sedentary activities factor (as a function of the questionnaire item “My father/mother or other adults let me play video and computer games as much as I want”). The final confirmatory model therefore did not support inclusion of the factor on permissiveness for sedentary activities. When stratified by gender, the data showed similar one-factor results. For boys the confirmatory model showed a RMSEA value of 0.092, SRMR of 0.0414, and an AGFI of 0.934, and for girls, it showed a RMSEA value of 0.023, SRMR of 0.0297, and an AGFI of 0.972.

**Table 4:** Individual item factor loadings in Turkish and English for the factor loadings for the Physical Activity Home Environment Scale (Child) showing two dimensions.

Turkish	English	Component	
		1	2
Ailem genellikle fiziksel olarak etkindir	My family is usually physically active.	.683	
Annem/babam veya diğer yetişkinler benimle yürüyüşe çıkarlar	My father/mother or other adults take walks with me.	.643	
Annem/babam veya diğer yetişkinlerin TV seyretmek yerine fiziksel olarak aktif olmam için uğraşırlar	My mother/father or other adults encourage me to be physically active instead of watching TV.	.636	
Annem/babam veya diğer yetişkinler hava güzel olduğu zaman dışarıda oynamam için uğraşırlar	My mother/father or other adults encourage me to play outside when the weather is good.	.590	
Yaşadığım yerin yakınında dışarıda oyun oynamak güvenlidir	It is safe to play outside close to my house.	.507	
Annem/babam veya diğer yetişkinler istediğim kadar TV seyretmeme izin verirler	My father/mother or other adults let me to watch TV as much as I want.		.833
Annem/babam veya diğer yetişkinler video ve bilgisayar oyunlarını istediğim kadar oynamama izin verirler	My father/mother or other adults let me play video and computer games as much as I want.		.796

## Discussion

In light of the rapidly rising childhood obesity rates in developing countries such as Turkey, validated and reliable measures are needed to effectively explore the multiple levels of factors that contribute to the obesity epidemic. The extent of the effect parental and child risk factors have on the development of childhood obesity in Turkey is currently not known. The present study is one of the first to focus on such factors. The main focus of COSA was to increase understanding of the individual and familial factors that are associated with childhood overweight and obesity and that might be relevant to parental support for various interventions in order to help inform future obesity interventions.

The Physical Activity Barriers Scale (parent) as a whole was found to have acceptable model fit statistics. Our results support the originally hypothesized two-factor structure—personal barriers and environmental barriers.[18] The Physical Activity Barriers subscales showed good internal consistency and poor-to-fair test-retest reliability (personal α = 0.79, ICC = 0.29, environmental α = 0.73, ICC = 0.59). For the personal barriers subscale, our results suggest some modification may be needed to improve the test-retest reliability in the Turkish context. For the environmental barriers, our results were somewhat stronger than the results other studies have reported for similar scales. In Germany, the relationship of the physical environment to physical activity levels was examined in 9–17 year-old male and female children.[39] Their findings for internal consistency were somewhat lower than ours, ranging from α = 0.42–0.64, while their ICC values for test-retest reliability were somewhat higher than our own (ICC = 0.59–0.74). Similar to our Physical Activity Barriers scale, an Australian study in grade 5 and 6 children evaluated the neighborhood physical environment, looking at the effect of factors such as accessibility, aesthetics, and safety on physical activity in these children.[40] They found poor to good internal consistency values (ranging from α = 0.43–0.65), which were lower than our findings, and good to excellent test-retest reliability values (ICC = 0.72–0.88), which were significantly higher than our results.

The Physical Activity Outcome Expectancies Scale (child) as a whole was also found to have good model fit statistics (RMSEA = 0.054, SRMR = 0.0545, AGFI = 0.916). Our results support the originally hypothesized two-factor structure—negative outcome expectancies and positive outcome expectancies.[32] The Physical Activity Outcome Expectancies subscales showed good internal consistency and fair test-retest reliability (negative outcome expectancies α = 0.77, ICC = 0.49; positive outcome expectancies α = 0.74, ICC = 0.56). Our results were similar to findings from other measures of outcome expectancies. For example, in the original Saunders “Beliefs” scale, which the Sherwood outcome expectancies scale is based on, researchers examined the beliefs of children regarding physical activity outcomes (both physical and social), and found α = 0.75 for the physical outcomes scale and α = 0.58 for the social outcomes scale.[41] The physical outcomes result is similar to our result, while the social outcomes α is somewhat lower. In Iran, the Trial of Activity in Adolescent Girls (TAAG) study found similar results to those of our study when looking at various psychosocial determinants of physical activity in 10^th^ grade adolescent girls.[42] One measure examined was decisional balance—child-perceived pros and cons to physical activity, similar to our Outcome Expectancies scale, and was found to have an α = 0.72.

The Physical Activity Home Environment Scale (child) showed good model fit statistics (RMSEA = 0.061, SRMR = 0.02332, and an AGFI = 0.968) as a one-factor model (support for physical activity). Our results did not support the originally hypothesized two-factor structure—support for physical activity and permissiveness for sedentary activities due to negative variance in the model.[33] Our results suggest that for the Physical Activity Home Environment scale, modification of the scale to reflect cultural differences may be needed to better adapt the constructs to the Turkish context. A one-factor model can be reasonably applied in research. However, it is recommended that future studies collecting data using this tool repeat the factor analysis to verify model structure and provide further evidence for the best model for the Turkish population. Other studies have shown stronger reliability in family social support measurements. In the GEMS study, the scale was validated only in urban African American girls ages 8–9 and showed α>0.86 for both subscales. The relationship of parental support to physical activity levels was also examined in Germany in 9-17-year-old male and female children.[39] Unlike our findings, they showed both strong internal consistency and test-retest reliability (α = 0.78, ICC = 0.83). Also in an Australian study, a home social environment scale was shown to have good internal consistency and excellent test-retest reliability (α = 0.73, ICC = 0.84), again showing stronger results in comparison to our parental support scale.[40] In Iran, the results from the TAAG study were closer to those of our study, perhaps indicating the presence of regional variability that needs to be further studied. When looking at family support for physical activity, the authors found good internal consistency and fair test-retest reliability (α = 0.72, r = 0.56).[42]

Cultural differences in the Turkish population compared to the population in which the scales were initially validated could have contributed to the lower reliability of both the Physical Activity Barriers and Home Environment scales. For example, cultural norms regarding physical activity or variations in digital access for children can differ between countries. Research has shown that rates of internet access for Turkish youth are still lower than Western levels, and that a significant gender divide in Internet access and use also exists.[43] In addition, the rate of participation in organized sports is low in Turkey, with a significant gender gap existing in this area as well.[44] The Turkish scales may need to be adapted to reflect these cultural differences. Our results provide general support for the direct adaptation and use of the Physical Activity Barriers, Physical Activity Outcome Expectancies, and the Physical Activity Home Environment scales in Turkey for measurement of psychosocial determinants of childhood obesity. Though some variability has been detected in comparison to studies from other countries, our confirmatory factor analysis demonstrates the applicability of these scales in Turkey with some small adaptations. Methodological research on such scales will expand scientific investigations on the contributions of sociocultural factors affecting obesity in Turkey, as well as allow for further comparisons with data from other countries.

Our study population consisted exclusively of fourth grade students and their parents from the city of Ankara, so our results may not be generalizable to other age groups or families living in rural areas of Turkey.

## Conclusions

Research using internationally validated scales will help to broaden our understanding of how social and cultural differences affect nutrition and physical exercise activity behaviors of children and families, which in turn contribute to childhood obesity. This will give rise to a deeper understanding of the obesity issues in Turkey with an eye toward more regionally tailored solutions rather than relying entirely on known evidence from non-Turkish settings.

The present study strengthens the research capacity for addressing obesity in Turkey. Results from future studies utilizing these scales will be able to directly inform intervention design and implementation to prevent or reduce childhood obesity and have the potential to initiate a cohort of families for longitudinal follow-up. This research will further contribute to the body of knowledge on childhood obesity in Turkey and the Eastern Mediterranean region as well as partnerships between researchers in the U.S. and Turkey, enhancing the research collaboration between the two countries and regions.

## Supporting information

S1 Dataset (ZIP)

## Abbreviations

COSA: Childhood Obesity Study of Ankara
COSI: Childhood Obesity Surveillance Initiative
RMSEA: root mean square error of approximation
SRMR: standardized root mean square residual
AGFI: adjusted goodness of fit index
ICC: intra-class correlation
SES: socio-economic strata
WHO: World Health Organization
PA: physical activity
US: United States
TAAG: Trial of Activity in Adolescent Girls
GEMS: Girls health Enrichment Multisite Study
